# Hirschsprung’s Disease and Associated Congenital Heart Defects: A Prospective Observational Study from a Single Institution

**DOI:** 10.3389/fped.2014.00099

**Published:** 2014-09-17

**Authors:** Giulia Tuo, Alessio Pini Prato, Maria Derchi, Manuela Mosconi, Girolamo Mattioli, Maurizio Marasini

**Affiliations:** ^1^Department of Pediatric Cardiology, Istituto Giannina Gaslini, Genoa, Italy; ^2^Department of Pediatric Surgery, Istituto Giannina Gaslini, Genoa, Italy; ^3^Department of Neuroscience, Ophthalmology, Rehabilitation, Genetics and Maternal-Infant Science (DINOGMI), University of Genoa, Genoa, Italy

**Keywords:** congenital heart disease, Hirschsprung’s disease, cardiac screening, neurocristopathy, echocardiography

## Abstract

**Objective:** To define the prevalence and characteristics of associated congenital heart diseases (CHDs) in patients with Hirschsprung’s disease (HSCR).

**Method:** All patients with a histological diagnosis of HSCR admitted to our hospital between January 2010 and December 2013 were included in this prospective observational study and underwent cardiovascular screening. Cardiac anatomy was assessed by a segmental echocardiographic approach. Measurements of aortic root and left ventricular dimensions, wall thickness, and function were obtained. CHDs requiring a percutaneous or surgical intervention were described as major heart diseases.

**Results:** One hundred thirty-three consecutive patients were enrolled at median age of 2.3 years. Eleven patients (8.3%) presented an associated heart disease. Moreover, five patients had mild dilatation of aortic root. Six out of 11 (4.5%) patients had a major CHDs requiring surgical repair.

**Conclusion:** Prevalence of associated CHDs was slightly higher than in previous papers, and mostly represented by septal defects. Four out of six patients with major heart disease had also a chromosomal anomaly. If we do not consider the subpopulation of patients with a chromosomal anomaly, cardiac defects were present in 3.8% of the patients. Based on these results, we suggest to perform routine echocardiogram in all Hirschsprung patients, with or without associated chromosomal syndromes.

## Introduction

Hirschsprung’s disease (HSCR) is the commonest congenital gut motility disorder and is characterized by the absence of ganglion cells in a variable length of the distal gut. This results in absent peristalsis in the affected bowel and it represents a frequent cause of functional intestinal obstruction especially in the newborn period (1–17). It occurs as a consequence of premature arrest of cranio-caudal migration of neural crest-derived neuroblasts (NCN) in the hindgut and is therefore described as a neurocristopathy (1, 17–19). Alterations of heterogenous genetic pathways involved in the enteric nervous system development may interfere with the colonization process of NCN and represents a primary etiology both for HSCR and for the anomalies of other organs and systems that often come together (13, 20–23).

According to literature, HSCR may be associated with a chromosomal abnormality or additional congenital anomalies in approximately 20% of cases (9, 16, 20, 21). Associated congenital heart diseases (CHDs) have a reported incidence of around 5%. However, there could be an underestimation of the reported incidence both because most of the studies were retrospective and focused on genotype rather than on phenotype determination, and because usually there is a mild or late symptoms onset with subsequent missed or delayed diagnosis (9, 20, 21).

The aim of our study was to assess the prevalence and the phenotype of associated CHDs in patients with HSCR.

## Materials and Methods

All consecutive HSCR patients admitted for further evaluation and/or surgical treatment to the G. Gaslini Institute between January 2010 and December 2013 were taken into account for this prospective observational study. Only those with a histological diagnosis of intestinal aganglionosis were included in the study and underwent cardiac screening (6). Exclusion criteria were uncertain HSCR diagnosis and/or refusal to sign the Informed Consent.

Enrolled patients and/or their parents were interviewed to collect personal and family history, length of aganglionosis, and any information regarding associated cardiac anomalies if already detected during previous phenotype screening.

Length-wise, four types of aganglionosis have been identified: short segment HSCR (S-HSCR) with aganglionosis extending up to the left descending colon, long segment HSCR (L-HSCR) with aganglionosis extending up to the right transverse/ascending colon, total colonic aganglionosis (TCA) with aganglionosis extending to the whole colon with or without small bowel involvement, and total intestinal aganglionosis (TIA) with aganglionosis extending over the jejunum with <20 cm of normoganglionic bowel (11).

For the purpose of our study, CHD was considered as “a gross structural abnormality of the heart or intrathoracic great vessels that is actually or potentially of functional significance,” as defined by Mitchell et al. in 1971. Moreover, CHD requiring a percutaneous or surgical intervention was described as *major* CHD (5, 7).

The cardiovascular screening included physical examination and an echocardiogram. Height, weight, and age were recorded in each patient as well as the non-invasive blood pressure measurement. Detailed echocardiograms were performed with a pulsed, continuous, and color-Doppler provided ultrasound system (iE33, Philips, Amsterdam, The Netherlands), using a 5 or 8 MHz transducer. Morphological variables were measured offline by two observers (Giulia Tuo and Maria Derchi) who used the so-called *z*-score for normalization of the cardiac structures dimensions to the body size (4, 15, 24).

Cardiac anatomy was routinely assessed by a segmental approach. Two-dimensional echocardiographic measurements included the end-sistolic diameter, respectively, of the aortic annulus, Valsalva sinuses, and ascending aorta. These dimensions were measured from the parasternal long-axis view with the “inner edge convention,” which uses the innermost bright edge reflection as a contour. The aortic root was considered dilated if the *z*-score was ≥2 and the degree of dilation was defined mild if the *z-*score was ≤3 (15). End-diastolic wall thickness and left ventricular internal dimensions were measured through the M-mode technique, which was performed also for calculating the shortening fraction. Diastolic ventricular function and valves function were assessed by both pulsed and continuous Doppler technique (2, 10, 12, 14).

For patients with a major CHD that required a trans-catheter or surgical treatment, we reported both clinical and echocardiographic details at the last follow-up examination.

Descriptive statistics were reported as percentages for categorical variables and as mean ± SD for continuous data. This study was approved by our institutional Ethics Committee and informed consent to view files, and echocardiographic reports was obtained by the parents.

## Results

During the 5 years study period, 133 consecutive HSCR patients were admitted to the Department of Surgery of the Gaslini Institute and underwent cardiac screening. Median age at enrollment was 2.3 years (range 3 months to 25 years). Male to female ratio was 4:1. Twenty patients (15%) had ultralong HSCR (either TCA or TIA), 9 (7%) had L-HSCR, and 104 (78%) had S-HSCR. Eight HSCR patients had also Down’s syndrome (6%).

Eleven patients (8.3%) presented an associated CHD of whom six patients were already aware at the time of enrollment (Table 1). Nine out of 11 were affected by the S-HSCR, 2/11 by the TCA. 5/11 (45%) were also affected by Down’s syndrome, and 1/11 by Turner’s syndrome. CHDs were mostly represented by septal defects (atrial, ventricular, or atrioventricular septal defect) and patent ductus arteriosus (Table 1). The family history was always unremarkable for any CHD or arrhythmia. Six patients (4.5%) presented a major CHD. Four out of six were also carrying a chromosomal abnormality, and they all required a cardiac surgical procedure. At a mean follow-up of 9 years (±5.8 years), they were all asymptomatic and without any residual cardiac defects on echocardiogram.

**Table 1:** **Details of HSCR patients with associated congenital heart diseases**.

Patient	Sex	Age (m)	Type of aganglionosis	Type of heart disease	Management	Syndrome
1	M	59	S-HSCR	Small OS ASD + small PDA	F-UP	
2	M	58	S-HSCR	Small OS ASD + small PM VSD	F-UP	Down
3	F	54	S-HSCR	Small OS ASD	F-UP	
4	M	51	S-HSCR	Large OS ASD + moderate PM VSD	SP	
5	F	309	S-HSCR	Large OP ASD	SP	Down
6	M	171	S-HSCR	Large OS ASD + moderate PM VSD	SP	Down
7	M	192	TCA	Large PM VSD	SP	Down
8	F	27	TCA	AC	SP	Turner
9	M	96	S-HSCR	Moderate size OS ASD	SP	
10	M	52	S-HSCR	Small PDA	F-UP	Down
11	M	107	S-HSCR	Small PDA	F-UP	

*M, male; F, female; m, months of life at time of cardiac screening; S-HSCR, short segment Hirschsprung’s disease; TCA, total colonic aganglionosis; OS, ostium secundum; OP, ostium primum; ASD, atrial septal defect; PM VSD, perimembranous ventricular septal defect; AC, aortic coarctation; PDA, patent ductus arteriosus; F-UP, follow up; SP, surgical procedure*.

In addition to the above-mentioned abnormalities, we observed mild dilatation of the aortic root in five patients. None of them had an associated chromosomal abnormality. They are all being followed up in the long term. None of the study population presented high blood pressure.

## Discussion

Hirschsprung’s disease is the main genetic cause of functional intestinal obstruction with an incidence of 1/5000 live births. It is characterized by the absence of the enteric ganglia in a variable length of the intestine, and it is the consequence of multiple gene interactions, which affect the ability of the enteric neural crest cells to differentiate in the developing gut (1, 13, 17–19, 22, 23).

Although HSCR occurs as an isolated phenotype in at least 70% of cases, a number of associated congenital anomalies and syndromes have been retrospectively reported. In particular, cardiac defects occur with a mean incidence of 5% of cases of HSCR and, among them, septation defects (atrial, ventricular, or atrioventricular septal defect) and conotruncal developmental defects are the most often described (8, 9, 16, 20, 21, 25, 26).

Among our study population, we had an overall detection rate of associated CHDs of 8.3%, which is significantly higher than prevalence reported by previous authors as the result of retrospective assessments of surgical series or of systematic literature reviews (13, 20, 23). We may speculate that this discrepancy of prevalence is due by the absence or mild severity of symptoms related to the associated CHDs in most HSCR patients that may lead to a misdiagnosis and/or a delayed diagnosis. In fact, 5/11 (45%) patients were diagnosed with an associated CHD only at the time of our cardiac screening.

However, we could confirm literature data regarding the type of detected CHDs, as they were mostly represented by septal defects. This is in accordance to the embryologic role of neural crest-derived stem cells in the development of both ENS and cardiac outflow septation (9, 13, 18, 20, 21). Noteworthy, none of our patients presented conotruncal heart defects whose pathogenesis is similarly related to the abnormal neural crest cell proliferation and migration. On the other hand, five patients showed a mild dilatation of the aortic root. To the best of our knowledge, this echocardiographic finding has never been reported before and we think that it would be worthy of further investigations for understanding if there is some correlation between this aortic wall anomaly and the multiple gene mutations involved in HSCR.

Six out of 11 patients (5.4%) had a major CHD of whom 4 had also an associated chromosomal abnormality. The overall incidence of Down’s syndrome in our study population was 6% (8/133). Five out of eight patients (62%) with HSCR and Down’s syndrome presented also an associated CHD that required a surgical intervention in three cases. In 2009, the 30-years retrospective nationwide survey carried out in Japan showed an increased incidence of Down’s syndrome in patients affected by HSCR and interestingly also an increased incidence of associated cardiac anomalies in the last 10 years (27). We believe it is worth noting that Down’s syndrome patients affected by HSCR presented a higher incidence of associated CHD than that previously reported in the overall Down’s syndrome population (mean 40%) (3, 27).

If we do not consider HSCR patients with Down’s syndrome, CHDs were still present in 3.8% of the study population, which means that the prevalence of CHDs in isolated HSCR patients is at least three times that of the general population (5, 7).

On the ground of these considerations, we suggest to investigate all HSCR patients, regardless of sex, length of aganglionosis, and associated syndromes, in order to exclude a possible associated cardiac malformation. In particular, we suggest to routinely perform an ECG and an echocardiogram in all patients admit for further evaluation and/or treatment of HSCR.

Our institution is known as a referral center in Italy for HSCR and other intestinal dysganglionoses that could lead to an intrinsic subsequent risk of selection BIAS. This concern is supported by the twofolds higher than expected incidence of ultralong forms of the disease (17.9%). Nonetheless, the prevalence of Down’s syndrome (6%) as well as the male to female ratio (4:1) are coherent with literature data and confirm that our series is representative of the whole HSCR population.

To conclude, our study demonstrated a higher than previously reported prevalence of associated cardiac anomalies in HSCR patients. A better knowledge of clinical phenotype prompted the implementation of an updated diagnostic algorithm aimed at improving the outcome and the quality of life of our patients. In fact, early diagnosis of associated CHDs allows prompt establishment of adequate treatment and prevention of possible complications related to a delayed diagnosis.

## Conflict of Interest Statement

The authors declare that the research was conducted in the absence of any commercial or financial relationships that could be construed as a potential conflict of interest.
